# Tools for Stools – Recognising and Managing Constipation on an Older Adult Inpatient Ward: A Quality Improvement Project

**DOI:** 10.1192/bjo.2025.10413

**Published:** 2025-06-20

**Authors:** Aishwarya Nathan, Romesa Khan, Modupe Odumosu, Louisa Bird, Margaret Ogbeide-Ihama

**Affiliations:** Oxleas NHS Trust, London, United Kingdom

## Abstract

**Aims:** Constipation is a common problem in psychiatric patients and can have serious consequences. Patients taking antipsychotics with anticholinergic properties are at higher risk of constipation. The risk is further increased in older adults due to reduced mobility and polypharmacy. To treat constipation, patients should have their bowel movements monitored and preventative laxatives considered. We faced similar challenges and this project was undertaken following a Serious Untoward Incident due to constipation related complications,

This quality improvement project aimed to increase detection, recording and management of constipation on an 18 bedded older adult inpatient ward. Thus, preventing complications like delirium, faecal impaction and bowel obstruction.

**Methods:** For this QI project, we used the Model for Improvement (MFI) framework, which involves defining the aim, measuring progress, and identifying changes that lead to improvement, along with the Plan-Do-Study-Act (PDSA) cycle. Bowel monitoring was initiated using the Bristol stool charts, with staff training and awareness sessions. Data collection was conducted for all 18 patients. In the second half of 2023, charts were integrated into MDT discussions, and further staff training was provided. In 2024, the bowel charts and Norgine risk assessment tool were used, a patient tutorial session provided, with data collected for all 18 patients and staff awareness assessed through questionnaires.

**Results:** Bowel monitoring and documentation significantly improved throughout the course of three audit cycles. In the first cycle, total number of days for all 17 patients that bowel movement was recorded was just 5 in 2 weeks and only 2 bowel charts uploaded onto the system. In the second cycle of 18 patients, 173 days recorded and 16 charts uploaded. In the third cycle of 18 patients, 151 days recorded with an average Norgine score of 7.056 and all charts uploaded. Thus, frequency of monitoring increased by 66.55% in the second cycle but then decreased by 8.73% in the third cycle although continuing to show improvement from the first.

**Conclusion:** This project aimed to improve bowel assessment in an inpatient mental health ward for patients on antipsychotics. Using the MFI framework, the data shows significant improvements in bowel monitoring and documentation over three cycles. Consistent uploading of bowel charts onto RIO and Norgine score assessments reflect a commitment to high standards in patient care. Improved bowel monitoring can reduce constipation, prevent complications and save costs through reduced laxative use and shorter hospital stays. This scientific approach underscores the importance of diligent monitoring in enhancing patient outcomes.

